# Psychometric Validation of a Spanish–Chilean Instrument for Assessing Public Attitudes Toward Childhood Stuttering: Construct Validity and Internal Consistency

**DOI:** 10.3390/children13040506

**Published:** 2026-04-03

**Authors:** Yasna Sandoval, Carlos Rojas, Francisco Novoa-Muñoz, Gabriel Lagos, Carla Figueroa, Álvaro Silva, Jaime Crisosto-Alarcón, Mauricio Alfaro-Calfullán

**Affiliations:** 1 Department of Health Rehabilitation Sciences, University of Bío-Bío, Chillán 4081112, Chile; ysandoval@ubiobio.cl (Y.S.); glagos@ubiobio.cl (G.L.); carlafig@gmail.com (C.F.); asilva@ubiobio.cl (Á.S.); jcrisosto@ubiobio.cl (J.C.-A.); 2 Department of Nursing, University of Bío-Bío, Chillán 4081112, Chile; fnovoa@ubiobio.cl

**Keywords:** childhood stuttering, public attitudes, stigma, psychometric validation, cross-cultural adaptation

## Abstract

**Highlights:**

**What are the main findings?**

- A culturally adapted Spanish–Chilean version of the POSHA-S Beliefs and Reactions scales showed strong construct validity and internal consistency after item refinement (14 and 11 items, respectively).
- Beliefs toward childhood stuttering were clustered into three independent factors (competence/normality, psychological causes, help/support). Reactions formed four factors (distant concern, personal concern, media sources, formal sources).

**What are the implication of the main finding?**

- The validated instrument enables reliable quantification of public stigma and misconceptions about childhood stuttering in Chile, particularly in educational settings.
- The results support the design of targeted school-based interventions and policies to reduce bullying, promote inclusion, and improve psychosocial outcomes for children and adolescents who stutter.

**Abstract:**

**Background/Objectives:** Stuttering is a neurodevelopmental disorder of speech fluency. It emerges most commonly between 2 and 5 years old, often causing social exclusion and stigma. In Latin America, cultural misconceptions regarding its causes exacerbate these psychosocial challenges. This study validated a culturally adapted instrument for Chile to measure public attitudes toward stuttering. The instrument provides a psychometrically sound method to assess and address stigma within educational and community settings. **Methods:** A total of 756 Chilean adults (mean age = 36.7 years, SD = 15.8; 64% women, 36% men) were recruited using stratified probability sampling to reflect the national demographics. Ethical approval and informed consent were obtained. The subsection underwent rigorous cross-cultural adaptation (translation, expert review, cognitive debriefing *n* = 30, pre-testing *n* = 50). Analysis employed polychoric matrices, EFA, CFA with WLSMV, and multiple reliability/validity indices. **Results:** Joint analysis showed poor fit (CFI = 0.72, RMSEA = 0.12), confirming independence. Beliefs (14 items): three-factor CFA fit excellent (CFI = 0.993, RMSEA = 0.034); factors: competence/normality (α = 0.85), psychological causes (α = 0.78), and help/support (α = 0.72). Reactions (11 items): four-factor fit adequate (CFI = 0.97, RMSEA = 0.043); factors: distant concern (α = 0.82), personal concern (α = 0.79), media sources (α = 0.75), and formal sources (α = 0.77). Validity was supported; bifactor models favored multidimensionality. **Conclusions:** The adapted subsection is psychometrically robust and effectively captures Chilean-specific attitudes toward childhood stuttering. It provides a reliable tool for quantifying public stigma and misconceptions, particularly in educational and school contexts, thereby supporting the design of targeted school-based policies and interventions to reduce bullying, promote inclusion, and safeguard the psychosocial well-being of children and adolescents who stutter.

## 1. Introduction

Stuttering is clinically defined as a neurodevelopmental disorder involving involuntary disruptions in speech fluency, such as repetitions, prolongations, and blocks. It emerges primarily between ages 2 and 5, affecting 8–11% of children worldwide during early childhood, with 1% persisting into adulthood [1,2].

The etiology is multifactorial, involving genetic, neurophysiological, and environmental factors, resulting in heterogeneous manifestations beyond disfluency, including secondary behaviors such as eye blinking, facial tension, or speech avoidance [3]. In children, these symptoms often coincide with critical developmental milestones, where language acquisition and social interaction are paramount, rendering stuttering a significant barrier to normative growth trajectories [4,5].

The psychosocial sequelae of stuttering are particularly acute during childhood and adolescence, periods characterized by heightened sensitivity to peer evaluation and self-identity formation. Longitudinal investigations reveal that children who stutter are predisposed to elevated levels of social anxiety, depressive symptomatology, and diminished self-esteem as early as primary school [6]. Early adverse encounters, including peer derision, mimicry, or social ostracism, engender internalized stigma, precipitating anticipatory anxiety and behavioral avoidance that may crystallize into enduring maladaptive patterns [7]. Meta-analytic evidence underscores bullying prevalence rates of 60–80% among school-aged children who stutter (primarily in primary and secondary education, roughly ages 6–18), exponentially higher than those of fluent peers, correlating with compromised academic engagement and protracted emotional morbidity [6,8]. These dynamics are amplified in educational settings, where verbal participation is integral to learning, potentially impeding cognitive and socioemotional development.

School contexts represent pivotal scenarios for the manifestation and mitigation of stuttering-related stigma in youth. Educators’ attitudinal frameworks profoundly modulate classroom experiences; erroneous attributions associating stuttering with cognitive deficits or emotional lability culminate in discriminatory practices, such as curtailed oral opportunities or attenuated expectations [9,10,11]. Empirical surveys of teachers indicate frequent inadvertent marginalization, wherein students who stutter are less frequently solicited for responses or interrupted mid-utterance, fostering disengagement and perpetuating silence [10,11]. Conversely, pedagogic settings integrating stuttering awareness and normalization protocols evince diminished bullying and augmented participation, as substantiated by randomized trials of concise educational modules [9]. This dichotomy illuminates the potential for informed instructional strategies to serve as protective buffers against psychosocial adversity in children.

Broader societal awareness constitutes a public health exigency for safeguarding child well-being, as advocated by entities such as the American Speech-Language-Hearing Association (ASHA) and the International Stuttering Awareness Day Committee [12]. Interventions as brief as 20–30 min have engendered sustained attitudinal improvements, attenuating stigmatizing conduct and bolstering empathy in pediatric cohorts [13,14,15]. Such initiatives not only insulate children who stutter from emotional trauma but also cultivate inclusive ecosystems wherein communicative variances are normalized, facilitating holistic development.

Cross-cultural variances in attitudes toward childhood stuttering are pronounced, modulated by sociocultural paradigms, historical legacies, and media depictions. Western milieus often exhibit moderate positivity, fortified by advocacy and empirical dissemination, yielding attitude scores proximate to neutrality [16]. In contrast, non-Western locales, including Asian and Middle Eastern regions, manifest negative orientations underpinned by convictions in volitional control or metaphysical etiology [17,18]. Latin America embodies a distinctive amalgamation: collectivist ethos, familial cohesion, and valorization of articulate expression escalate stuttering’s perceived encumbrance in children [19]. Regional studies report pervasive attributions to emotional weakness, parental failure, or culturally mediated supernatural and fear-based explanations, thereby amplifying blame and eroding compassionate responses, particularly within educational contexts where verbal proficiency is highly valued [19].

In Chile, these sociocultural undercurrents are accentuated by indigenous, colonial, and contemporary amalgamations, engendering environments where fluent discourse is esteemed in juvenile social and academic domains. Contemporary studies reveal that substantial adult segments—exceeding 60% in select samples—construe children who stutter as intrinsically “nervous” or “diffident,” proffering inefficacious counsel such as deceleration [10,19,20]. Pedagogues, despite benevolent predispositions, often articulate deficits in preparedness to aid pupils who stutter, thereby precipitating unwitting bias [10]. Furthermore, Chile’s burgeoning immigrant and plurilingual juvenile populace—propelled by migrations from Venezuela, Haiti, and Peru—confronts aggravated impediments, with stigma intersecting linguistic disparities in educational milieus [21]. These confluences exacerbate internalized stigma, manifesting in social retraction and scholastic underperformance during adolescence [10].

Within this sociocultural context, the paucity of culturally attuned instruments has impeded methodical scrutiny of childhood stuttering stigma in Chile. Absent dependable metrics, delineating stigma magnitudes, discerning predominant stereotypes in scholastic contexts, or appraising awareness initiatives for youth remains elusive. To redress this, the present inquiry adapted a subsection from an established survey on public attitudes, concentrating on scales appraising beliefs and reactions pertinent to childhood stuttering perceptions. This subsection, encompassing 35 items, furnishes scores mirroring attitudes impinging on school dynamics for children and adolescents.

Worldwide adaptations evince the subsection’s adaptability. European iterations manifest elevated reliability (α > 0.80) and neutral scores, modulated by urbanization and pedagogy [16,22]. North American and Oceanic inquiries report mildly adverse scores with sturdy consistency, evincing intervention responsiveness [23,24]. Asian and Middle Eastern accommodations necessitate calibrated modifications for notions like “nervousness,” yielding pessimistic scores and underscoring affective or ethereal origins [17,25,26]. Latin American endeavors, encompassing Puerto Rican Spanish, proffer auspicious reliability and scores analogous to U.S. benchmarks yet unveil locale-specific attributions. Incipient Chilean utilizations intimate gender and socioeconomic mediators, with females and elevated-education cohorts manifesting affirmative dispositions [10].

Within this sociocultural context, the paucity of culturally attuned instruments has impeded methodical scrutiny of childhood stuttering stigma in Chile. Absent dependable metrics, delineating stigma magnitudes, discerning predominant stereotypes in scholastic contexts, or appraising awareness initiatives for youth remains elusive. To redress this, the present inquiry adapted a subsection from an established survey on public attitudes toward stuttering—the Public Opinion Survey of Human Attributes–Stuttering (POSHA-S)—concentrating specifically on the Beliefs and Reactions scales. We focused exclusively on these two dimensions because they directly capture the core attitudinal components most relevant to childhood stuttering stigma in educational and social contexts: cognitive attributions (Beliefs) and emotional/behavioral responses (Reactions). The remaining POSHA-S scales (e.g., Self, Accommodating/Helping, Knowledge/Source, and the overall stuttering rating) were excluded as they address more general or personal attributes less specific to public perceptions of children and adolescents who stutter in school settings—the primary focus of this validation study. This subsection, encompassing 35 items originally, furnishes scores mirroring attitudes impinging on school dynamics for children and adolescents.

## 2. Materials and Methods

This study adopted an instrumental design focused on psychometric evaluation, adhering to methodological standards for scale validation in psychological measurement [27,28]. The approach emphasized cultural sensitivity in adaptation, rigorous sampling, and advanced statistical techniques to ensure the Chilean Spanish POSHA-S’s applicability in diverse Chilean contexts. All procedures complied with ethical guidelines, promoting transparency and replicability.

### 2.1. Participants and Procedure

A total of 756 Chilean adults (mean age = 36.7 years, SD = 15.8; 64% women, 36% men) were recruited using stratified probability sampling to reflect the national demographics. A total of 55% of participants had completed high school, and 45% had completed university studies. The participants were drawn from the Ñuble and Bío-Bío regions to represent Chile’s central-southern diversity. Inclusion criteria were native Spanish speakers aged 18–65 with no self-reported history of stuttering or related disorders in themselves or their immediate families, in order to isolate general public attitudes. Responses with >10% missing data or evident response patterns (e.g., straight-lining) were excluded.

Data collection took place between April and July 2023. Surveys were administered in person at public venues, such as community centres, markets, and workplaces. The response rate was 72%, with non-respondents primarily citing time constraints as the reason for not responding. Participants received no incentives beyond an informational debriefing on stuttering awareness. Ethical approval was granted by the University of Bío-Bío Institutional Review Board (protocol UBB-2023-045, 15 March 2023), ensuring compliance with the Declaration of Helsinki. Informed consent, obtained electronically or in print, detailed the study purpose, anonymity, voluntary participation, and data usage. No generative artificial intelligence was utilized in the design, collection, analysis, or interpretation, thus preserving methodological integrity.

### 2.2. Instrument

The POSHA-S, a 46-item questionnaire, assesses multifaceted attitudes toward stuttering, yielding an OSS and subscale scores [29]. This study targeted its primary scales: Beliefs (18 items, C1–C18, probing cognitive representations, causal attributions, competence perceptions) and Reactions (17 items, R1–R17, evaluating emotional, behavioral, and self-regulatory responses). Items employ 5-point Likert scales (1 = strongly disagree to 5 = strongly agree), with some reversed for polarity balance.

Adaptation followed cross-cultural guidelines [30]: (1) Forward translation by two independent bilingual speech-language pathologists familiar with Chilean dialects; (2) synthesis into a preliminary version; (3) backward translation by two separate experts; (4) expert committee review (*n* = 5: linguists, clinicians, cultural anthropologists) to resolve discrepancies and incorporate regional expressions (e.g., “stuttering” contextualized with everyday scenarios); (5) cognitive debriefing interviews conducted with 30 demographically diverse pilot participants to evaluate item clarity, relevance, and cultural appropriateness, and this phase resulted in minor rephrasing, particularly for items addressing etiological beliefs; and (6) a subsequent pre-test involving 50 respondents to confirm adequate comprehension and an acceptable administration time (approximately 15 min).

### 2.3. Data Analysis

Given ordinal data, analyses commenced with polychoric correlation matrices to account for non-normality [28,31]. Scales were analyzed independently based on theoretical and empirical evidence of distinct domains [29]. Exploratory factor analysis (EFA) used principal axis factoring with oblique rotation (promax) for correlated factors, retaining based on parallel analysis, eigenvalues > 1, and interpretability. Kaiser–Meyer–Olkin (KMO) and Bartlett’s sphericity tested sampling adequacy. Item depuration criteria were loadings < 0.40, cross-loadings > 0.30, conceptual misalignment, or Heywood cases indicating specification issues.

Confirmatory factor analysis (CFA) via WLSMV estimation in Mplus v8.3 validated EFA-derived structures. Fit thresholds were CFI/TLI ≥ 0.95 (excellent), ≥0.90 (adequate); RMSEA ≤ 0.05 (excellent), ≤0.08 (adequate); and SRMR ≤ 0.08 [32,33]. Modification indices guided refinements if theoretically justified. Reliability metrics included ordinal alpha (α), McDonald’s omega (ω), and composite reliability (CR), with ≥0.70 acceptable. Convergent validity included AVE ≥ 0.50; discriminant, with interfactor r < 0.85 and square root of AVE > interfactor correlations. Bifactor models evaluated general factor dominance, ECV > 0.60 and ωh > 0.70, favoring unidimensionality [34,35]. In cases of conflicting indices, priority was given to CFI and TLI (incremental fit indices) over RMSEA, consistent with recommendations for ordinal data analyzed with WLSMV estimation, where RMSEA can be overly sensitive to sample size and model complexity [28,32,33]. SRMR was used as a supplementary absolute fit measure. The final dataset contained no missing responses (0% missingness after exclusion of incomplete or invalid cases). Consequently, no missing data handling procedures, such as Full Information Maximum Likelihood (FIML), were required or applied. All models were estimated directly using Weighted Least Squares Mean and Variance adjusted (WLSMV) estimation, which is the recommended and most appropriate method for ordered-categorical indicators with complete data [28]. Descriptive statistics (means, SDs) contextualized factor scores, with assumptions checked for multivariate normality and outliers.

## 3. Results

The Section 3 of this instrumental study provides a detailed psychometric evaluation of the Spanish–Chilean adaptation of the POSHA-S, focusing on its two primary scales: Beliefs and Reactions. The analyses were conducted using polychoric correlation matrices to account for the ordinal nature of the items, with exploratory factor analysis (EFA) and confirmatory factor analysis (CFA) employed to assess construct validity. Reliability was evaluated through ordinal alpha, McDonald’s omega, and composite reliability, while convergent and discriminant validity were examined via Average Variance Extracted (AVE) and interfactor correlations. The findings underscore the scales’ independence, refined factorial structures following item depuration, and robust psychometric properties, supporting the instrument’s utility in measuring public attitudes toward stuttering in a Chilean context. Below, the results are described in subsections, with tables summarizing key metrics and placeholders for the graphical representations mentioned in the document.

### 3.1. Joint Psychometric Analysis of the Beliefs and Reactions Scales

The study initially explored the feasibility of modeling the POSHA-S as an integrated multidimensional instrument by combining items from the Beliefs scale (C1–C18) and Reactions scale (R1–R17). This approach was based on a preliminary hypothesis that the scales might represent interrelated dimensions of a global attitude toward stuttering, potentially allowing for a unified latent construct. However, empirical evidence consistently demonstrated that such integration was not psychometrically viable, highlighting the distinct nature of the two domains.

In the exploratory phase, the analyses yielded unstable structures. Factors formed from idiosyncratic item combinations, with no cross-scale integration. Loadings clustered almost entirely within each scale. This indicated clear structural independence between the cognitive Beliefs domain and the emotional–behavioral Reactions domain. Therefore, the scales capture related but distinct aspects of attitudes, rather than a single overarching construct.

Confirmatory models further confirmed these issues, yielding systematically deficient fit indices across multiple estimations. Specifically, the Comparative Fit Index (CFI) and Tucker–Lewis Index (TLI) remained below 0.80, with values indicative of poor model data correspondence. The Root Mean Square Error of Approximation (RMSEA) consistently exceeded 0.10, signaling a high level of approximation error, while the Standardized Root Mean Square Residual (SRMR) reached elevated levels, reflecting substantial discrepancies between observed and model-reproduced correlations. These indices collectively indicate unacceptable model adjustment, according to both classical and contemporary criteria [32,33]. This poor joint fit indicates that Beliefs and Reactions do not form a single unified attitudinal construct in the Chilean context. For applied users (e.g., educators, school psychologists, and speech-language therapists), this separation is practically meaningful: it suggests that interventions should address cognitive misconceptions (Beliefs) and emotional/behavioral responses (Reactions) as distinct targets, particularly within school settings where both types of attitudes influence bullying, inclusion, and psychosocial support for children who stutter.

Additional technical challenges compounded the analysis, including non-convergent solutions under Weighted Least Squares Mean and Variance Adjusted (WLSMV) and maximum likelihood (ML) estimators, negative residual variances (known as Heywood cases), and non-positive definite polychoric correlation matrices. Such problems are typically associated with model misspecification, excessive conceptual heterogeneity, or inadequate handling of items with opposing directionality [28]. In this case, the presence of inverted and polarized items across scales exacerbated these issues. For the Beliefs scale, negative content (e.g., items related to concealment or blame) coexisted with positive formulations on competence and social normality. Similarly, in Reactions, socially desirable behaviors were mixed with potentially inappropriate responses (e.g., impatience or mockery). This mixture led to inconsistent correlation patterns and the emergence of artificial factors, a phenomenon well-documented in the psychometric literature [36].

Taken together, these results offer robust evidence that the Beliefs and Reactions scales do not constitute a single global latent construct. Instead, they represent conceptually interrelated but empirically distinguishable domains. This outcome aligns with theoretical expectations in attitude measurement, where cognitive and affective components often warrant separate treatment. Consequently, the decision was made to conduct independent psychometric analyses for each scale, a methodological choice grounded in principles of construct validity. This separation not only improves model identifiability but also enhances the interpretability of the instrument for practical applications in assessing attitudes toward stuttering.

### 3.2. Psychometric Results for the Beliefs Scale

#### 3.2.1. Exploratory Factor Analysis (EFA)

The EFA was performed on the original 18-item Beliefs scale using polychoric correlations to accommodate the ordinal item format. The global Kaiser–Meyer–Olkin (KMO) measure of sampling adequacy was 0.56, which is marginal but still within the minimally acceptable range (≥0.50) for proceeding with factor analysis. This value was deemed sufficient given the highly significant Bartlett’s test of sphericity (χ^2^ = 8717.22, df = 153, *p* < 0.001), which confirmed patterned relationships among items and indicated that the correlation matrix was not an identity matrix and thus appropriate for factor extraction.

Examination of individual Measure of Sampling Adequacy (MSA) values revealed low adequacy for items linked to extreme or non-scientific etiological explanations, such as supernatural or viral causes. These items exhibited weak factorial loadings, cross-saturations, and inconsistent contributions to the latent structure. Initial solutions also detected Heywood cases, pointing to conceptual heterogeneity and specification challenges commonly encountered in complex attitudinal scales [28].

To address these issues, depuration was guided by empirical (e.g., loadings and communalities) and theoretical criteria. Items C1 and C5 were inverted to align their negative wording with the predominant positive direction of the construct. Subsequently, items C9, C10, C14, and C12 were progressively eliminated due to poor psychometric performance and limited conceptual coherence with the scale’s core. This elimination pattern is consistent with prior research highlighting the instability of extreme etiological beliefs in stuttering attitude instruments [13,37].

Following depuration, parallel analysis and exploratory fit criteria endorsed a three-factor solution. This structure was conceptually interpretable, with an average complexity close to 1.1, signifying a simple configuration devoid of significant cross-loadings.

#### 3.2.2. Confirmatory Factor Analysis (CFA)

**Figure 1:**
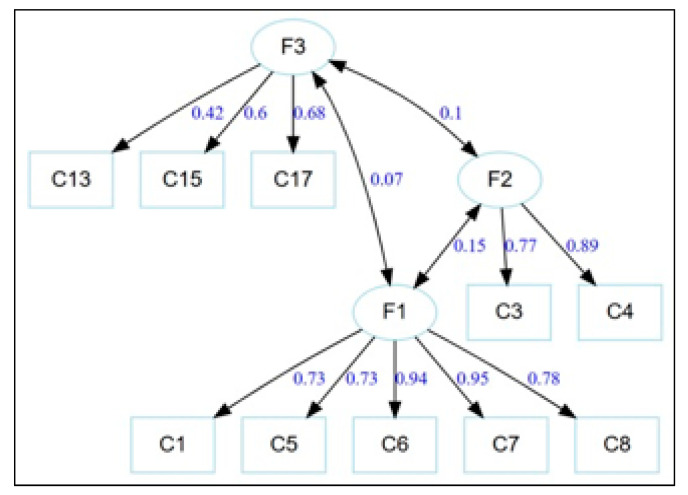
Confirmatory factor analysis (CFA) model of the Beliefs scale.

The three-factor structure derived from EFA was tested with CFA under WLSMV estimation. The correlated model showed excellent adjustment to the data (CFI = 0.993; TLI = 0.990; RMSEA = 0.034; SRMR = 0.056), surpassing thresholds for good and excellent structural quality [32,33]. For more details, see Figure 1.

All standardized factorial loadings were statistically significant (*p* < 0.001) and ranged from moderate to high (λ = 0.43–0.95). Interfactor correlations were low to moderate (r = 0.07–0.15), providing support for discriminant validity among the identified dimensions. A supplementary bifactor model, which posits a general factor alongside specific factors, showed high total reliability (ωt = 0.88) but moderate hierarchical reliability (ωh = 0.62) and an Explained Common Variance (ECV) of 0.40. These indices suggest that the general factor is not sufficiently dominant to warrant a single total score, favoring a multidimensional interpretation [34,35].

Interpretations of the factors are based on the final CFA model, ensuring empirical validation, stability, and replicability [28]. Factor denominations consider standardized loading patterns and observed interfactor correlations.

Factor 1, labeled “Beliefs about the competence, normality and social agency of people who stutter” (items C1, C5, C6, C7, C8), evaluates beliefs regarding the functional and social competence of individuals who stutter, encompassing perceptions of their ability to lead normal lives, form social relationships, and perform adequately in academic and occupational settings. The literature identifies these beliefs as central to stuttering stigma, directly impacting social acceptance and perceived opportunities [37,38]. The factor reflects the extent to which respondents attribute—or deny—autonomy, competence, and social legitimacy to people who stutter. High scores indicate inclusive, less stigmatizing beliefs, while low scores denote deficit-focused, blame-oriented, or concealment-promoting views. Theoretically, this dimension connects to classic stigma and social attitudes toward communicative disabilities [3,38]. Psychometrically, it forms the scale’s most robust core, with strong loadings and adequate internal consistency.

Factor 2, “Beliefs about the psychological or personal causes of stuttering” (items C3, C4), aggregates beliefs attributing stuttering to emotional or dispositional traits, such as nervousness, shyness, or fear. This represents a personality-centered conceptualization, one of the most common erroneous explanations for stuttering, associated with stigmatization processes and negative communicative expectations [13]. Comprising only two items, the factor nonetheless displays elevated loadings and acceptable reliability, consistently capturing the “psychologization” of stuttering as a reduction to individual emotional characteristics.

Factor 3, “Beliefs about the types of help and social support available for people who stutter” (items C13, C15, C17), assesses beliefs related to sources of help and support, including learning-based explanations and the legitimacy of aid from peers, close contacts, or other stutterers. Prior studies show these beliefs influence support provision willingness, treatment efficacy expectations, and social responsibility perceptions [13]. The dimension portrays stuttering as amenable to social, relational, and learning processes beyond exclusively medical or individualistic frameworks. It links to contemporary perspectives emphasizing social support and communicative contexts in coping with stuttering [39]. Psychometrically, the factor exhibits moderate internal consistency, as anticipated given the domain’s conceptual heterogeneity, yet it adds a theoretically relevant and differentiated element to the scale. Table 1 summarizes the factor structure, including items, loadings, and reliability metrics for the Beliefs scale.

### 3.3. Psychometric Results for the Reactions Scale

#### 3.3.1. Exploratory Factor Analysis (EFA)

The initial EFA on the 17-item Reactions scale revealed low global reliability (α ≈ 0.47) and reduced average inter-item correlation (r^−^ ≈ 0.05), indicating marked content heterogeneity. Exploratory solutions were unstable and lacked interpretability, regardless of the retained factor number. Initial depuration produced an intermediate version with partial improvements (KMO ≈ 0.64; α ≈ 0.59), but structures remained non-parsimonious. Subsequent elimination of item R5 decisively clarified the internal organization.

The final 11-item version demonstrated acceptable sampling adequacy (KMO = 0.64) and a significant Bartlett test. A four-factor solution optimized fit (RMSEA = 0.040; TLI = 0.946; RMSR = 0.02), with the lowest Bayesian Information Criterion (BIC), low-to-moderate interfactor correlations, and average complexity near 1.1.

#### 3.3.2. Confirmatory Factor Analysis (CFA)

**Figure 2:**
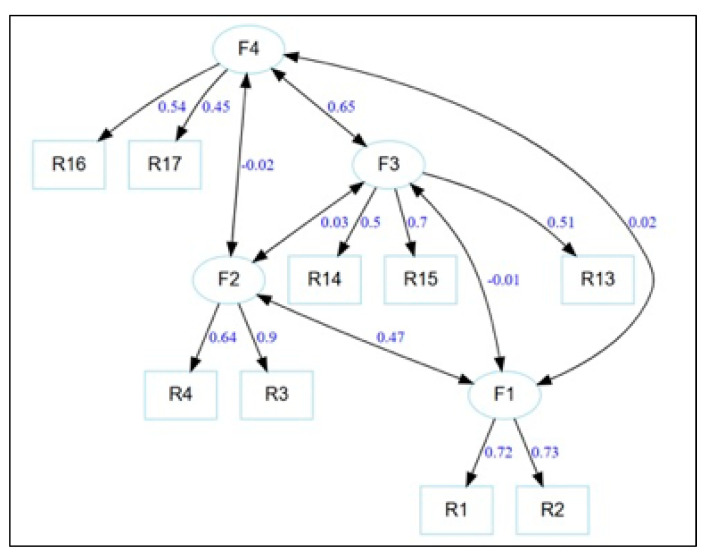
Confirmatory factor analysis (CFA) model of the Reactions scale.

CFA of the correlated four-factor model showed adequate fit (χ^2^(21) = 50.84, *p* < 0.001; CFI = 0.97; TLI = 0.95; RMSEA = 0.043; SRMR = 0.035). All loadings were significant and moderate to high. Bifactor analyses confirmed the absence of a dominant general factor (ECV ≈ 0.03; ωh ≈ 0.04), advising against total scores and endorsing multidimensionality [34,35]. For more details, see Figure 2.

Factor interpretations are based exclusively on the final CFA model, derived from depurated items, representing the validated, stable latent structure of affective, behavioral, and informational reactions to stuttering [28,33].

Factor 1, “Distant social reactions of concern toward stuttering” (items R1 [“my doctor”], R2 [“my neighbor”]), groups anticipatory concern reactions when stuttering occurs in non-intimate social environments. Items reflect affective unease tied to functional roles or external relationships. Conceptually, this links to implicit social attitudes viewing stuttering as problematic in instrumental contexts, a key public stigma element [37,38]. Psychometrically, it features elevated loadings and adequate internal coherence, consistently capturing distant social affective reactions.

Factor 2, “Personal and family reactions of concern to stuttering” (items R3 [“my brother/sister”], R4 [“I”]), represents concern when stuttering affects personal or close family realms, including self. Unlike Factor 1, it involves direct, self-referential emotional engagement. This pattern matches the literature distinguishing general social attitudes from personal reactions, where emotional impact heightens with proximity [3,13]. Psychometrically, the factor shows clear structure despite limited items, delineating a specific personal affective dimension.

Factor 3, “Media and digital sources of information about stuttering” (items R13 [television/radio/movies], R14 [journals/books], R15 [internet]), aggregates media/digital knowledge sources. It reflects how mass media and platforms nourish attitudes and reactions via social representations. The literature notes the media’s pivotal role in stereotype building, affecting normalization or stigma perpetuation [13]. Moderate internal consistency is expected from channel variety, yet it captures a differentiated knowledge socialization dimension.

Factor 4, “Formal and professional sources of information about stuttering” (items R16 [formal education], R17 [healthcare professionals]), represents formal, academic, and professional information sources linked to structured education and health experts. This reflects an institutionalized, evidence-based knowledge approach. Theoretically, it aligns with models stressing health literacy and professionals’ stigma-reduction role [39]. Psychometrically, clear loadings and coherent structure justify its independent status. Table 2 summarizes the factor structure and psychometric metrics for the reactions scale

## 4. Discussion

The psychometric validation of the Chilean Spanish adaptation of the subsection from the POCHA-S yields compelling evidence of its construct validity and internal consistency, particularly when applied to the measurement of public attitudes toward childhood and adolescent stuttering. This study, grounded in instrumental design principles for psychological measurement [27,40,41], employed polychoric correlations to accommodate the ordinal nature of the items, alongside exploratory factor analysis (EFA) and confirmatory factor analysis (CFA) with Weighted Least Squares Mean and Variance Adjusted (WLSMV) estimation. The findings affirm the instrument’s multidimensionality, with refined factorial structures following item depuration, excellent to adequate model fits, significant and substantial loadings, and robust reliability metrics, including ordinal alpha, McDonald’s omega, and composite reliability. Convergent validity was supported by Average Variance Extracted (AVE) values, while discriminant validity was evidenced by consistently low interfactor correlations. Bifactor models further underscored the absence of a dominant general factor, advocating for subscale-specific interpretations rather than unidimensional scoring. These outcomes not only validate the adapted subsection for use in Chilean contexts but also contribute meaningfully to cross-cultural attitude research on stuttering, aligning with the International Project on Attitudes Toward Human Attributes (IPATHA) framework [29]. More importantly, the results hold particular relevance for understanding and addressing the stigma experienced by children and adolescents who stutter, a population especially vulnerable to negative public perceptions during critical developmental periods.

The initial attempt to model the adapted subsection as an integrated instrument by jointly analyzing the Beliefs (C1–C18) and Reactions (R1–R17) scales revealed profound psychometric challenges, providing strong empirical substantiation for their conceptual and structural independence. Exploratory models exhibited marked instability, with idiosyncratic factor groupings confined to intra-scale items and no evidence of cross-domain integration. This pattern strongly suggests that cognitive representations captured by the Beliefs scale and affective–behavioral responses captured by the Reactions scale operate as distinct latent constructs rather than facets of a unified global attitude. This lack of integration was further evidenced by systematically deficient fit indices, non-convergent solutions, and classic indicators of model misspecification (e.g., Heywood cases and non–positive definite correlation matrices). These technical artifacts are indicative of model misspecification, excessive conceptual heterogeneity, and inadequate handling of item polarity [28,36]. Theoretically, this separation is consistent with dual-process models of attitudes, which posit that cognitive and affective components may diverge, particularly in the context of stigmatized conditions, such as childhood stuttering [13]. In Chilean cultural environments, where collectivist norms place a high value on verbal fluency and social harmony [19], this distinction becomes especially meaningful. Beliefs may capture entrenched misconceptions that shape how adults perceive and interact with children who stutter in school settings, while Reactions reflect situational emotional responses that can directly influence peer dynamics and bullying behaviors. For example, targeted teacher training programs could address misconceptions captured by Beliefs (e.g., attributing stuttering to nervousness) while fostering supportive Reactions (e.g., reducing interruptions or mockery) to decrease bullying and promote inclusive verbal participation in the classroom.

### 4.1. Beliefs Scale

For the Beliefs scale, iterative depuration refined the original 18 items to 14, systematically eliminating items tied to extreme or non-scientific etiological explanations (C9, C10, C14, C12) due to weak loadings, cross-saturations, and conceptual incoherence. This refinement is consistent with prior validations that have highlighted the cultural and psychometric volatility of such items in attitudinal scales focused on stuttering [13,37]. The resulting three-factor EFA solution, supported by parallel analysis, produced a parsimonious structure (average complexity ≈ 1.1), while the confirmatory analysis showed excellent adjustment (CFI = 0.993; TLI = 0.990; RMSEA = 0.034; SRMR = 0.056). All standardized loadings were statistically significant (*p* < 0.001) and ranged from moderate to high (λ = 0.43–0.95), with low to moderate interfactor correlations (r = 0.07–0.15) providing clear support for discriminant validity. Bifactor indices (ωt = 0.88; ωh = 0.62; ECV = 0.40) indicated that while total reliability was high, the general factor lacked sufficient dominance to justify a single composite score, reinforcing the need for multidimensional interpretation [34,35].

Factor 1 (items C1, C5, C6, C7, C8) encapsulates perceptions of functional and social competence, including the perceived capacity of children who stutter to lead normal lives, establish peer relationships, and perform adequately in academic settings. This dimension constitutes a core component of public stigma, as deficit-oriented beliefs directly undermine social acceptance and limit opportunities for children during critical developmental windows [13,37,38,42]. In collectivist Latin American contexts, where communicative fluency is closely tied to social integration and group harmony, this factor may be particularly salient in shaping teacher expectations and peer interactions in school environments [19]. Factor 2 (items C3, C4) captures the persistent tendency to attribute stuttering to emotional or dispositional traits such as nervousness, shyness, or fear. This psychologization represents one of the most common and stigmatizing misconceptions about childhood stuttering, as it shifts responsibility onto the child and fosters blame rather than support [13,37]. Despite comprising only two items, the factor exhibited elevated loadings and acceptable reliability, demonstrating its ability to consistently measure this specific dimension of erroneous causal attribution. Factor 3 (items C13, C15, C17) evaluates beliefs in relational and learning-based sources of support, including the legitimacy of aid from peers, family, and others who stutter. This dimension aligns closely with contemporary ecological models of disability, particularly the International Classification of Functioning, Disability and Health (ICF), which emphasizes environmental facilitators over purely individual deficits [39,43]. Moderate internal consistency is expected given the conceptual breadth of the domain, yet the factor contributes a theoretically meaningful and practically relevant perspective on how adults conceptualize support for children who stutter in school and community settings.

### 4.2. Reactions Scale

The Reactions scale, initially characterized by marked heterogeneity (α ≈ 0.47; average inter-item correlation ≈ 0.05), required substantial depuration to achieve structural clarity, resulting in an 11-item version. The four-factor EFA solution optimized fit (RMSEA = 0.040; TLI = 0.946; RMSR = 0.02), and the confirmatory model yielded adequate adjustment (CFI = 0.97; TLI = 0.95; RMSEA = 0.043; SRMR = 0.035). All loadings remained significant and moderate to high, while bifactor indices (ECV ≈ 0.03; ωh ≈ 0.04) provided decisive evidence against a dominant general factor, reinforcing the appropriateness of multidimensional scoring [34,35].

Factor 1 (items R1, R2) reflects anticipatory concern when stuttering is observed in non-intimate social roles, such as those of teachers, doctors, or neighbors. This dimension embodies a form of public stigma that is particularly relevant to childhood contexts, as distant concern can translate into reluctance to engage with children who stutter in formal educational or extracurricular settings [13,37,38]. Factor 2 (items R3, R4) captures heightened emotional involvement when stuttering affects close relationships or the self, a response pattern that intensifies as proximity increases and is especially pertinent to sibling dynamics or parental attitudes toward a child’s condition [3]. Factors 3 (items R13, R14, R15) and 4 (items R16, R17) differentiate pathways through which attitudes are formed. The media factor highlights the role of mass and digital media in perpetuating stereotypes about childhood stuttering, which can be particularly influential during adolescence when media consumption peaks [37,38]. The formal sources factor underscores the importance of evidence-based health literacy and professional guidance in counteracting stigma, offering a pathway for school-based interventions led by educators and clinicians [39].

Comparatively, the factorial structures identified in this Chilean adaptation refine and extend previous global validations of the POSHA-S. European adaptations typically demonstrate high reliability and neutral to slightly positive attitude scores [16], while Asian versions reveal more negative orientations requiring cultural modifications [17]. The Chilean findings suggest moderately negative attitudes, shaped by the psychologization of stuttering and media influences, aligning with broader Latin American patterns. Viewed through a childhood and adolescent lens, these findings underscore the central role of adult attitudes in shaping school climates, peer interactions, and long-term developmental trajectories for children who stutter.

### 4.3. Advantages, Projections, and Future Directions

The adapted subsection offers several methodological and applied advantages, particularly in the context of childhood and adolescent stuttering research. Analyzing the scales separately reduces risks of misspecification from forced unidimensional models. This leads to better model adjustment and clearer structures. The depuration process enhances parsimony and reduces respondent burden (e.g., 14-item Beliefs scale) while preserving the majority of variance and conceptual coverage. The multifactorial design facilitates granular insights. Beliefs subscales enable precise identification of cognitive targets for educational interventions aimed at teachers and peers, while Reactions subscales allow for targeted strategies addressing emotional and behavioral responses in school settings. Culturally, the adaptation incorporates Chilean linguistic and idiomatic nuances, ensuring ecological validity in a collectivist society where supernatural and emotional attributions to stuttering remain prevalent [19]. Integration into the IPATHA database supports cross-cultural benchmarking, advancing comparative research on how public attitudes influence childhood stigma across diverse sociocultural contexts [44]. Practically, the instrument empowers evidence-based programs designed to reduce bullying and promote inclusion in schools, such as brief awareness interventions shown to produce measurable improvements in peer attitudes and classroom participation among children and adolescents [37,38]. It also provides a foundation for policy development supporting immigrant and multilingual youth, who face intersecting forms of stigma in educational environments [21].

Projections for the instrument’s utility are substantial. Longitudinal application could enable tracking of attitude shifts in response to school-based anti-stigma programs, offering empirical evidence of their effectiveness in reducing psychosocial burdens for children who stutter. The subsection could also serve as an outcome measure in intervention trials targeting specific attitudinal dimensions, such as countering psychologization through myth-busting campaigns or leveraging formal information sources through teacher training [37,38]. Future directions should include multisite replications across different Chilean regions to enhance generalizability, formal testing of measurement invariance across demographic groups (e.g., parents, teachers, adolescents), and convergent validation with child-specific attitude measures or behavioral observation protocols. Incorporating digital and social media sentiment analysis could enrich the Reactions scale, particularly given adolescents’ high exposure to online representations of stuttering. Cross-cultural meta-analyses integrating IPATHA data may further elucidate regional patterns in Latin America, informing tailored anti-stigma policies that prioritize the developmental needs of children and youth.

### 4.4. Limitations

Despite its strengths, several limitations warrant cautious interpretation of the findings. Methodologically, the item depuration process, although empirically and theoretically justified, may have resulted in the exclusion of culturally salient beliefs, particularly etiological attributions that could be relevant to childhood perceptions of stuttering. The presence of two-item factors (e.g., Beliefs Factor 2) limits statistical robustness, even though their high loadings and conceptual coherence support retention.

The absence of a dominant general factor in bifactor models precludes the use of total scores, complicating direct comparisons with unidimensional attitude instruments. Cultural specificity to Chilean Spanish may constrain generalizability to other Spanish-speaking populations, underscoring the need for formal measurement invariance testing across linguistic and national contexts.

Additional limitations include the regional concentration of the sample in the Bío-Bío area, which may not fully capture national diversity, and the lack of test–retest reliability, criterion-related validity, and sensitivity-to-change analyses. Finally, reliance on adult self-report may introduce social desirability bias, particularly when assessing attitudes toward stigmatized conditions in children, where reported attitudes may not fully reflect actual behaviors. The absence of direct assessment of attitudes among children and adolescents themselves further limits conclusions regarding peer-level stigma processes. Future studies could strengthen these brief subscales by developing and validating additional items for the two-item factors (e.g., psychological causes, distant concern, personal concern), thereby improving their reliability and enabling more robust independent use.

## 5. Conclusions

The Spanish–Chilean adaptation of the POSHA-S subsection demonstrates robust construct validity and internal consistency, with the Beliefs and Reactions scales operating as independent multidimensional instruments for the assessment of attitudes toward childhood stuttering. Joint modeling proved psychometrically untenable due to poor model fit and conceptual heterogeneity, thereby justifying the separate analysis of both scales. The Beliefs scale yielded a stable three-factor structure (competence, psychological causes, and social support), refined to 14 items and exhibiting excellent confirmatory fit, whereas the Reactions scale revealed a four-factor configuration (distant and personal concern; media-based and formal information sources), achieving adequate model fit following depuration to 11 items. Bifactor analyses confirmed the absence of a dominant general factor, supporting the use of subscale-specific scores rather than a unidimensional total score. Collectively, these findings validate the adapted subsection as a psychometrically sound and culturally appropriate tool for investigating public attitudes toward childhood and adolescent stuttering in Chile, enabling the quantification of stigma in school contexts and informing the development of ICF-aligned interventions aimed at reducing bullying and fostering inclusive educational environments. These findings underscore the urgent need for school-based policies and interventions in Chile that directly target public misconceptions and emotional reactions toward childhood stuttering, with the aim of reducing bullying, fostering inclusive classrooms, and improving the psychosocial outcomes of children and adolescents who stutter.

## Figures and Tables

**Table 1:** Factor structure and psychometric metrics for the beliefs scale.

* Factor Beliefs	Item	Standardized Loading (λ)	Reliability Metrics (α Ordinal, ω, Composite Reliability)	AVE	Interfactor Correlations
**1**	C1, C5, C6, C7, C8	0.43–0.95	α = N/A (not specified), ωt = 0.88 (total), ωh = 0.62	N/A	r = 0.07–0.15
**2**	C3, C4	0.43–0.95	As above	N/A	As above
**3**	C13, C15, C17	0.43–0.95	As above	N/A	As above

* Note: Specific per-factor reliability and AVE are not detailed in the document; they are aggregated from the bifactor model.

**Table 2:** Factor structure and psychometric metrics for the reactions scale.

Factor Reactions	Item	Standardized Loading (λ)	Reliability Metrics (α Ordinal, ω, Composite Reliability)	AVE	Interfactor Correlations
**1**	R1, R2	Moderate to high	α = N/A, ωh = 0.04	N/A	Low to moderate
**2**	R3, R4	Moderate to high	As above	N/A	As above
**3**	R13, R14, R15	Moderate to high	As above	N/A	As above
**4**	R16, R17	Moderate to high	As above	N/A	As above

Note: Specific per-factor metrics aggregated from bifactor analysis.

## Data Availability

The raw data supporting the conclusions of this article will be made available by the authors upon request. The data are not publicly available because sensitive participant data is handled.
